# Supplementary material for: Long term declines in the functional diversity of sharks in the coastal oceans of eastern Australia
Source: Commun Biol. 2024 May 21;7:611. doi: 10.1038/s42003-024-06308-0 (PMC11109089; doi:10.1038/s42003-024-06308-0)
Supplement: Supplementary file 2 — Supplementary Information [file 42003_2024_6308_MOESM2_ESM.docx]

**Supplementary information**


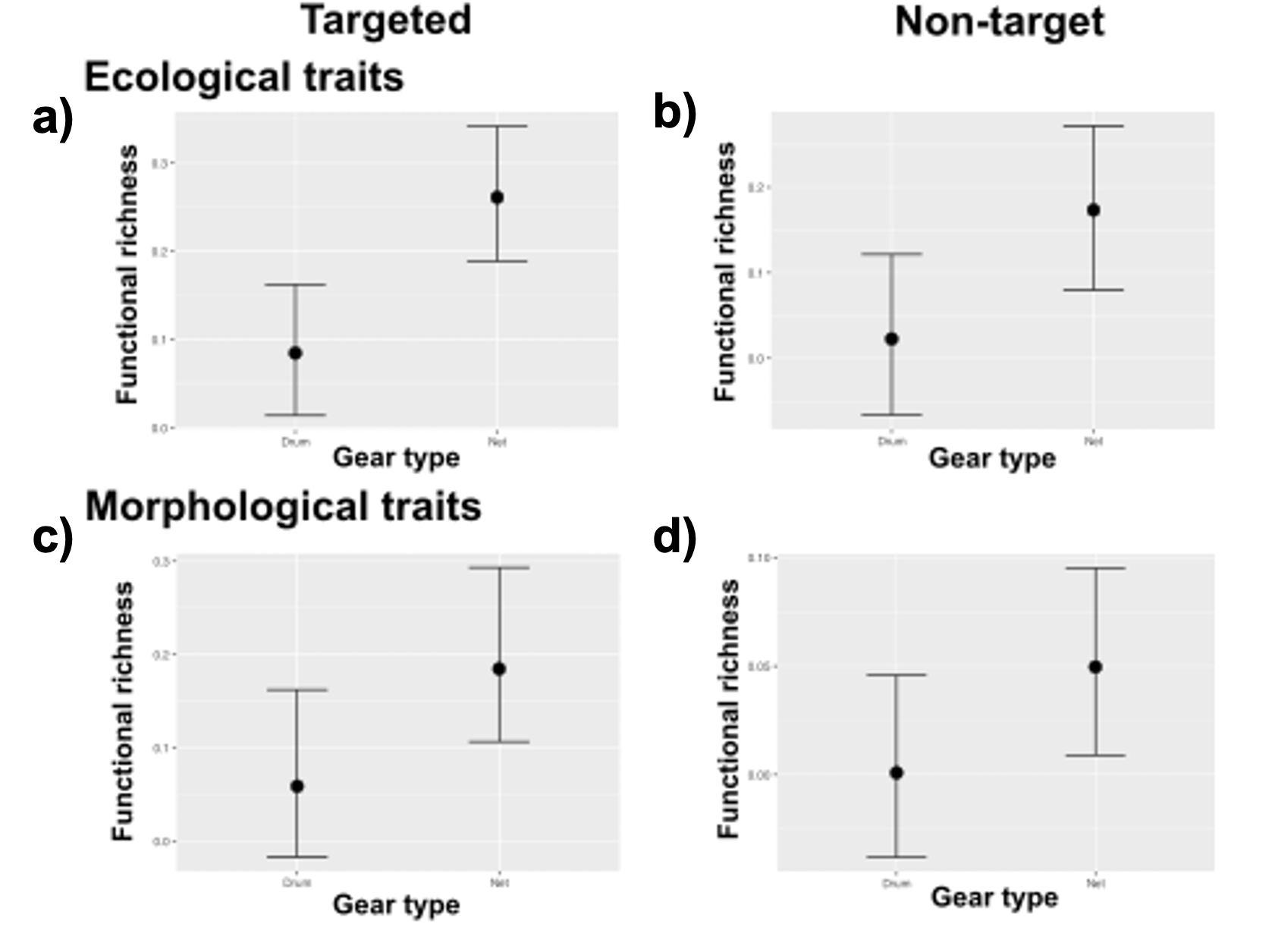


Figure S1. Bayesian Generalised Additive Mixed Models conditional effects plots on functional metrics for each gear type with the random grouping effect included for (a, c) ecological and morphological traits of apex sharks and (b, d) ecological and morphological traits of non-target species.


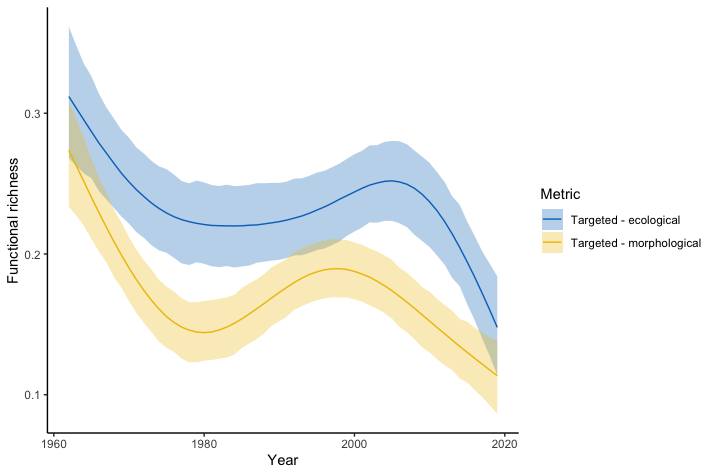


Figure S2. Bayesian Generalised Additive Mixed Models on the ecological and morphological functional richness of targeted apex sharks with whaler and hammerhead shark groups not combined. This is highlighted to show the influence of improved identification after a modification to the program in 1996.


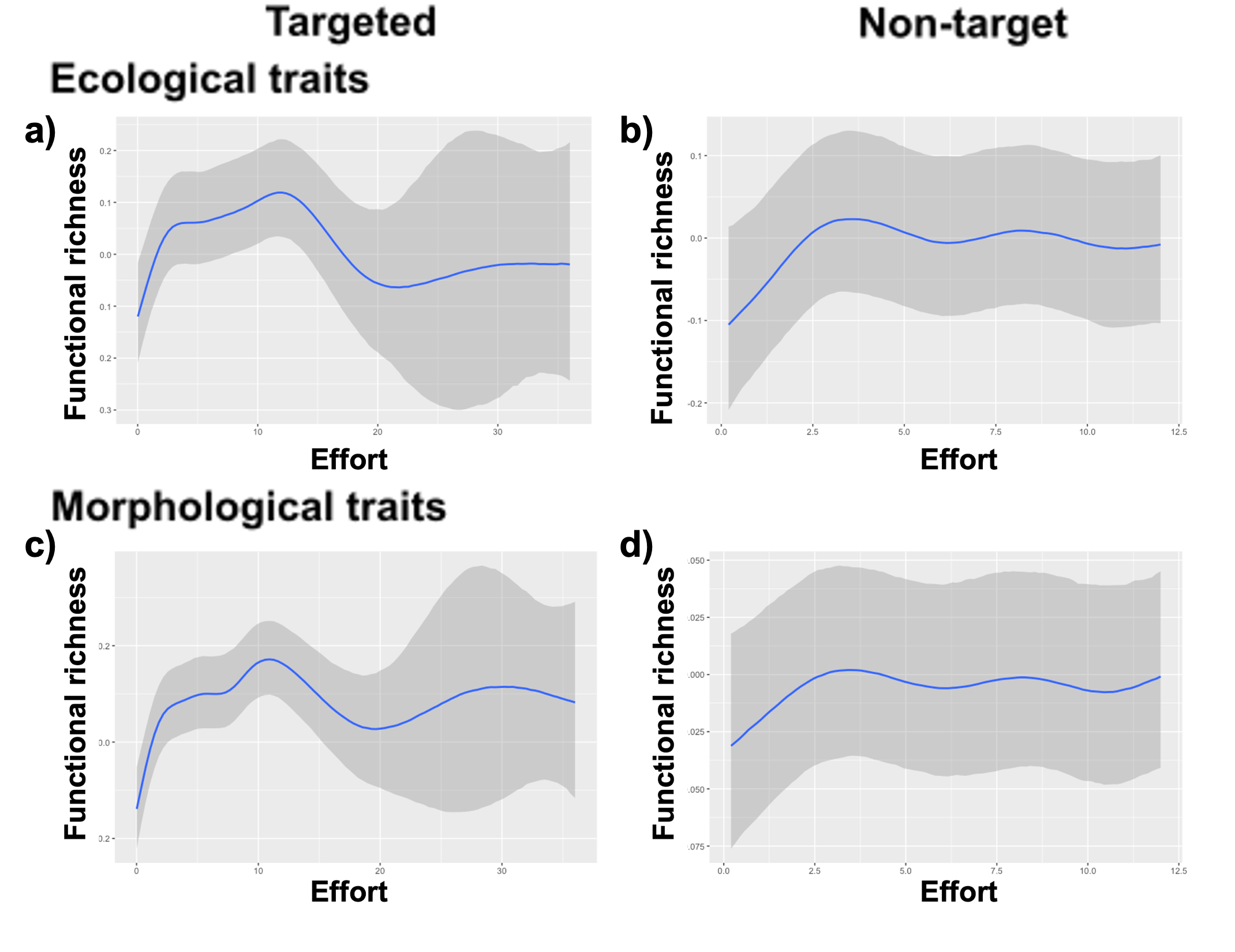


Figure S3. Bayesian Generalised Additive Mixed Models conditional effects plots on functional metrics against effort with the random grouping effect included for (a, c) ecological and morphological traits of apex sharks and (b, d) ecological and morphological traits of non-target species.

Table S1. Traits used to calculate functional richness come from two different categories, ecological and morphological. Here, we highlight the link to functioning for each trait, the categories used and the definition of that trait. References highlight previous studies using these traits and the location of the trait information.

| **Trait** | **Link to function** | **Categories** | **Definition** | **References** |
| --- | --- | --- | --- | --- |
| *Ecological traits* | |  |  | ^1-4^ |
| Habitat preference | The foraging activities of fish allows them to act as ecosystem engineers or modify food webs, having a functional impact on the ecosystem. | Reef associated | Mostly associated with rocky or coral reefs |  |
|  |  | Coastal pelagic | Shelf habitats but mostly feeds in the water column |  |
|  |  | Coastal benthic | Shelf habitats but mostly feeds in the benthos |  |
|  |  | Oceanic pelagic | Oceanic habitats but mostly feeds in the water column |  |
|  |  | Oceanic benthic | Oceanic habitats but mostly feeds in the benthos |  |
| Feeding group | Fishes have the potential to control the abundance of other organisms through predation and the overall structure of the ecosystem, and this is dependent on what they feed on. | Megafauna feeding | Feeds on large bodied species (e.g. turtles, seals, whales, dolphins, sharks) |  |
|  |  | Large-bodied fish feeding | Feeds predominantly on larger teleosts, rays and small sharks |  |
|  |  | Small-bodied fish feeding | Feeds predominantly on small teleosts |  |
|  |  | Invertebrate feeding | Feeds predominantly on invertebrates (e.g. benthic invertebrates and cephalopods) |  |
| Movement scale | The scale of movement a species migrates across indicates the spatial distribution of their functional impact. | 0-100 km | Small movements, typically site attached |  |
|  |  | 100-500 km | Moderate movements |  |
|  |  | 500 km | Large migratory patterns |  |
| *Morphological traits* | |  |  |  |
| Maximum size | Size is related to food intake and to their impact on the food web | Continuous | The maximum size of an individual species (regardless of sex) | ^1-8^ |
| Head length | Larger heads indicate fish able to handle larger prey | Continuous | The distance from the front of the upper lip to the operculum or first gill (as a proportion of total length) |  |
| Eye diameter | Fish with larger eyes can detect prey better | Continuous | The diameter of the eye (as a proportion of head length) |  |
| Pre-orbital length | An indicator of the types of habitats, and prey items, a fish might feed on | Continuous | The length from the snout to the anterior of the eye (as a proportion of total length) |  |
| Trophic level | Provides information on the level at which a species feeds in a food web | Continuous | An approximation of the position it occupies in a food web |  |
| Body depth | The depth of a fishes body relates to where they will feed | Continuous | The depth of the body (as a proportion of total length) |  |
| Teeth | Tooth shape is involved in food processing and may vary across diets and even between modes of capture | Triangular | A triangle shaped tooth, typically includes serrations |  |
|  |  | Angular |  |  |
|  |  | Grinding plate | A grinding plate typically used for invertebrate feeding |  |

**Table S2.** Calculated functional uniqueness and functional specialization values for each species

| **Species** | **Scientific name** | **Functional Uniqueness** | **Functional Specialization** |
| --- | --- | --- | --- |
| Australian blacktip | *Carcharhinus tilstoni* | 0.241 | 0.583 |
| Blacktip reef whaler | *Carcharhinus melanopterus* | 0.103 | 0.387 |
| Blue shark | *Prionace glauca* | 0.638 | 0.613 |
| Bronze whaler | *Carcharhinus brachyurus* | 0.000 | 0.005 |
| Bull whaler | *Carcharhinus leucas* | 0.011 | 0.029 |
| Common blacktip whaler | *Carcharhinus limbatus* | 0.100 | 0.352 |
| Dusky whaler | *Carcharhinus obscurus* | 0.163 | 0.449 |
| Graceful whaler | *Carcharhinus amblyrhynchoides* | 0.241 | 0.610 |
| Great hammerhead | *Sphyrna mokarran* | 0.410 | 0.884 |
| Grey nurse shark | *Carcharias taurus* | 0.199 | 0.319 |
| Grey reef whaler | *Carcharhinus amblyrhynchos* | 0.114 | 0.425 |
| Hammerhead shark | *Sphyrna spp.* | 0.498 | 0.585 |
| Long nose whaler | *Carcharhinus brevipinna* | 0.076 | 0.231 |
| Mako shark | *Isurus oxyrinchus* | 0.462 | 0.753 |
| Milk shark | *Rhizoprionodon acutus* | 0.324 | 0.650 |
| Pigeye whaler | *Carcharhinus amboinensis* | 0.076 | 0.231 |
| Sandbar whaler | *Carcharhinus plumbeus* | 0.586 | 0.498 |
| Scalloped hammerhead | *Sphyrna lewini* | 0.317 | 0.556 |
| School shark | *Galeorhinus galeus* | 1.000 | 0.856 |
| Sharptooth shark | *Negaprion acutidens* | 0.421 | 0.456 |
| Silky whaler | *Carcharhinus falciformis* | 0.212 | 0.321 |
| Spot-tail whaler | *Carcharhinus sorrah* | 0.500 | 0.668 |
| Tiger shark | *Galeocerdo cuvier* | 0.752 | 1.000 |
| Whaler | *Carcharhinidae spp.* | 0.022 | 0.000 |
| White shark | *Carcharodon carcharias* | 0.414 | 0.757 |
| White-cheek shark | *Carcharhinus dussumieri* | 0.318 | 0.596 |
| Whitetip reef shark | *Triaenodon obesus* | 0.164 | 0.432 |
| Australian sharpnose shark | *Rhizoprionodon taylori* | 0.253 | 0.108 |
| Barracuda | *Sphyraena barracuda* | 0.420 | 0.350 |
| Barramundi | *Lates calcarifer* | 0.001 | 0.000 |
| Batfish | *Platax teira* | 0.001 | 0.002 |
| Black cobia | *Rachycentron canadum* | 0.252 | 0.106 |
| Blind shark | *Brachaelurus waddi* | 0.336 | 0.519 |
| Blue groper | *Achoerodus viridis* | 0.420 | 0.329 |
| Bonito | *Sarda australis* | 0.420 | 0.217 |
| Bull ray | *Myliobatis australis* | 0.005 | 0.012 |
| Catfish | *Neoarius graeffei* | 0.001 | 0.012 |
| Conga eel | *Conger sp.* | 0.000 | 0.010 |
| Cownose ray | *Rhinoptera neglecta* | 0.841 | 0.632 |
| Devilray | *Mobula sp.* | 0.674 | 0.538 |
| Eastern Shovelnose ray | *Aptychotrema rostrata* | 0.000 | 0.010 |
| Fossil shark | *Hemipristis elongata* | 0.505 | 0.775 |
| Giant shovelnose ray | *Glaucostegus typus* | 0.170 | 0.246 |
| Giant trevally | *Caranx ignobilis* | 0.000 | 0.000 |
| Green sawfish | *Pristis zijsron* | 0.172 | 0.251 |
| Grey carpet shark | *Chiloscyllium punctatum* | 0.000 | 0.010 |
| Queensland groper | *Epinephelus lanceolatus* | 0.840 | 0.813 |
| Gummy shark | *Mustelus antarcticus* | 0.421 | 0.281 |
| Herring | *Elops machnata* | 0.420 | 0.299 |
| Jewfish | *Argyrosomus japonicus* | 0.000 | 0.010 |
| Kingfish | *Seriola lalandi* | 0.253 | 0.106 |
| Mackeral | *Scomberomorus commerson* | 0.337 | 0.583 |
| Manta ray | *Mobula alfredi* | 0.760 | 0.680 |
| Marlin | Makaira mazara | 0.508 | 0.780 |
| Narrow sawfish | *Anoxypristis cuspidata* | 0.254 | 0.261 |
| Nervous shark | *Carcharhinus cautus* | 0.336 | 0.518 |
| Port Jackson shark | *Heterodontus portusjacksoni* | 0.000 | 0.000 |
| Queenfish | *Scomberoides lysan* | 0.000 | 0.001 |
| Queensland sawfish | *Pristis clavata* | 0.170 | 0.247 |
| Reticulate whipray | *Himantura uarnak* | 0.254 | 0.260 |
| Australian salmon | *Polydactylus macrochir* | 0.255 | 0.259 |
| Shark ray | *Rhina ancylostoma* | 0.589 | 0.459 |
| Slit eye shark | *Loxodon macrorhinus* | 0.756 | 0.710 |
| Snapper | *Chrysophrys auratus* | 0.000 | 0.001 |
| Speartooth shark | *Glyphis glyphis* | 0.423 | 0.806 |
| Stingaree | *Trygonoptera testacea* | 0.420 | 0.348 |
| Sweetlip | *Diagramma pictum* | 0.000 | 0.001 |
| Swordfish | *Xiphias gladius* | 0.508 | 0.857 |
| Tasselled wobbegong | *Eucrossorhinus dasypogon* | 0.000 | 0.010 |
| Tawny shark | *Nebrius ferrugineus* | 0.420 | 0.369 |
| Toadfish | *Lagocephalus sceleratus* | 0.000 | 0.011 |
| Tropical sawshark | *Pristiophorus delicatus* | 0.000 | 0.011 |
| Tuna | *Thunnus albacares* | 0.420 | 0.623 |
| Turrum | *Turrum fulvoguttatus* | 0.000 | 0.001 |
| Wahoo | *Acanthocybium solandri* | 0.337 | 0.583 |
| Weasel shark | *Hemigaleus australiensis* | 0.421 | 0.292 |
| Whale shark | *Rhincodon typus* | 1.000 | 1.000 |
| White-cheek shark | *Aetobatus narinari* | 0.588 | 0.750 |
| White-spotted eagle | *Rhynchobatus australiae* | 0.423 | 0.397 |
| White-spotted guitarfish | *Eusphyra blochii* | 0.170 | 0.244 |
| Winged hammerhead | *Stegostoma tigrinum* | 0.422 | 0.824 |
| Zebra shark | *Carcharhinus tilstoni)* | 0.421 | 0.288 |

**References**

1 Cachera, M. & Le Loc'h, F. Assessing the relationships between phylogenetic and functional singularities in sharks (Chondrichthyes). *Ecology and Evolution* **7**, 6292-6303 (2017). <https://doi.org:https://doi.org/10.1002/ece3.2871>

2 Froese, R. & Pauly, D. FishBase 2000: concepts, design and datasources. (Los Baños, Laguna, 2023).

3 Stuart-Smith, R. D. *et al.* Integrating abundance and functional traits reveals new global hotspots of fish diversity. *Nature* **501**, 539-542 (2013). <https://doi.org:10.1038/nature12529>

4 Villéger, S., Brosse, S., Mouchet, M., Mouillot, D. & Vanni, M. J. Functional ecology of fish: current approaches and future challenges. *Aquatic Sciences* **79**, 783-801 (2017). <https://doi.org:10.1007/s00027-017-0546-z>

5 Dolbeth, M., Vendel, A. L., Pessanha, A. & Patrício, J. Functional diversity of fish communities in two tropical estuaries subjected to anthropogenic disturbance. *Marine Pollution Bulletin* **112**, 244-254 (2016). <https://doi.org:https://doi.org/10.1016/j.marpolbul.2016.08.011>

6 Leitão, R. P. *et al.* Disentangling the pathways of land use impacts on the functional structure of fish assemblages in Amazon streams. *Ecography* **41**, 219-232 (2018). <https://doi.org:doi:10.1111/ecog.02845>

7 Carscallen, W. M. A., Vandenberg, K., Lawson, J. M., Martinez, N. D. & Romanuk, T. N. Estimating trophic position in marine and estuarine food webs. *Ecosphere* **3** (2012).

8 Wainwright, P. C. & Richard, B. A. Predicting patterns of prey use from morphology of fishes. *Environ Biol Fish* **44**, 97-113 (1995). <https://doi.org:10.1007/bf00005909>
