# Supplementary material for: Long term declines in the functional diversity of sharks in the coastal oceans of eastern Australia
Source: Commun Biol. 2024 May 21;7:611. doi: 10.1038/s42003-024-06308-0 (PMC11109089; doi:10.1038/s42003-024-06308-0)
Supplement: Supplementary file 3 — Description of Additional Supplementary Files [file 42003_2024_6308_MOESM3_ESM.docx]

**Description of Additional Supplementary Files**

**File name:** Supplementary Data 1

**Description:** The functional traits of targeted and non-targeted coastal fish species using a series of ecological and morphological traits that quantify each species functional niche (e.g. function provided, feeding position and movement scale), conservation status (e.g. IUCN category) and morphological variation (e.g. maximum total length).
